# The Future of Kawasaki Disease Diagnosis: Liquid Biopsy May Hold the Key

**DOI:** 10.3390/ijms25158062

**Published:** 2024-07-24

**Authors:** Kasturi Markandran, Kristine Nicole Mendoza Clemente, Elena Tan, Karan Attal, Qiao Zhi Chee, Christine Cheung, Ching Kit Chen

**Affiliations:** 1 Department of Paediatrics, Yong Loo Lin School of Medicine, National University of Singapore, Singapore 119228, Singapore; kas_mark@nus.edu.sg (K.M.);; 2 School of Medicine, Royal College of Surgeons in Ireland, D02 YN77 Dublin, Ireland; 3 Division of Cardiology, Department of Paediatrics, Khoo Teck Puat–National University Children’s Medical Institute, National University Health System, Singapore 119228, Singapore; 4 Lee Kong Chian School of Medicine, Experimental Medicine Building, 59 Nanyang Drive, Nanyang Technological University, Singapore 636921, Singapore; 5 Institute of Molecular and Cell Biology, 61 Biopolis Drive, Proteos, Singapore 138673, Singapore

**Keywords:** Kawasaki disease, circulating endothelial cells, cell-free DNA, diagnostics, translational medicine, liquid biopsy

## Abstract

Kawasaki disease (KD) is a febrile illness characterised by systemic inflammation of small- and medium-sized blood vessels, which commonly occurs in young children. Although self-limiting, there is a risk of developing coronary artery lesions as the disease progresses, with delay in diagnosis and treatment. Unfortunately, the diagnosis of KD continues to remain a clinical dilemma. Thus, this article not only summarises the key research gaps associated with KD, but also evaluates the possibility of using circulating endothelial injury biomarkers, such as circulating endothelial cells, endothelial microparticles and vascular endothelial cell-free DNA, as diagnostic and prognostic tools for KD: a “liquid biopsy” approach. The challenges of translating liquid biopsies to use in KD and the opportunities for improvement in its diagnosis and management that such translation may provide are discussed. The use of endothelial damage markers, which are easily obtained via blood collection, as diagnostic tools is promising, and we hope this will be translated to clinical applications in the near future.

## 1. Introduction

### 1.1. Kawasaki Disease

Kawasaki disease (KD) is an acute systemic inflammatory syndrome of unknown aetiology, which predominantly affects children below six years of age [1]. It is an acute, typically self-limiting febrile illness that induces systemic vasculitis, mainly affecting small- and medium-sized arteries throughout the body, and it is one of the most prevalent vasculitis syndromes in childhood. Coronary arteries have the highest predilection to inflammation and in untreated KD, coronary artery lesions (CALs) such as aneurysmal dilatation can occur in 25–30% of patients [1,2]. This is the dominant pathological change, accounting for the major long-term morbidity and mortality of this disease [3].

It is recognised that there is a higher incidence of KD in Asians and Pacific Islanders (~250/100,000 children) compared to the U.S. and Europeans (~25/100,000 children) [1,2,3,4], affecting females and males at a ratio of 1:1.5. The recurrence rate is 3.5% in Asians and Pacific Islanders and 1.7% in the U.S. [1]. This is of particular concern, as recurrent disease increases the risk of developing cardiac sequelae [5]. The development of CALs could result in significant cardiovascular complications, such as myocardial ischaemia from coronary artery thrombosis and stenosis, and even fatality from acute myocardial infarction [6]. Therefore, there is much ongoing interest, and indeed some urgency, to improve the diagnosis and treatment of this disease.

The primary treatment of KD involves intravenous immunoglobulin (IVIg) with high-dose acetylsalicylic acid (aspirin) for their anti-inflammatory and immunomodulatory effects [1]. IVIg treatment reduces the occurrence of CAL in KD to a mere 5% when administered promptly [7]. As a result of these long-term cardiac sequelae, which cannot be fully mitigated, KD is one of the leading causes of acquired heart disease in children globally [8].

**Figure 1:**
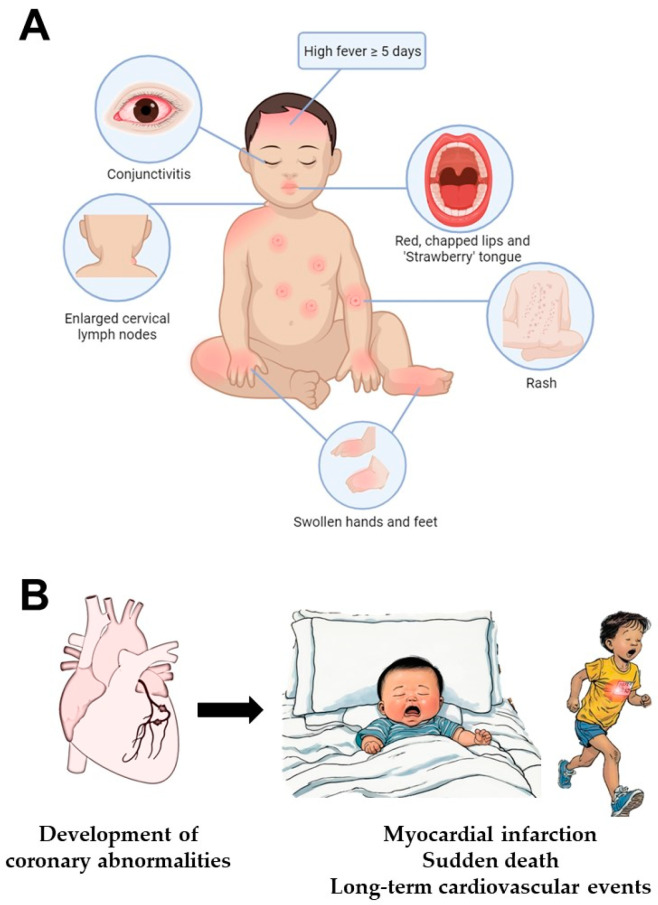
(**A**) Principal clinical features of Kawasaki disease. (**B**) Complications of Kawasaki disease.

In the absence of a confirmatory test, the diagnosis of KD rests on the identification of principal clinical features (Figure 1) and the exclusion of other known conditions, which may present similarly. A key feature of KD is fever persisting for at least five days. To establish the diagnosis of classic KD, fever is accompanied by at least four of five principal clinical features, including (1) extremity changes (redness and swelling of the hands and feet), (2) oral mucosal changes (redness and cracking of lips and tongue [“strawberry tongue”]), (3) polymorphous skin rash, (4) non-suppurative conjunctivitis (red eyes) and (5) cervical lymphadenopathy (lymph node swelling ≥ 1.5 cm in diameter) [9]. In the case where patients fall short of fulfilling the criteria for classic KD, other laboratory and imaging tests may support the diagnosis of incomplete KD [1]. It is challenging to diagnose KD, as the clinical characteristics may not develop concurrently. The symptoms are very similar to other common childhood illnesses, such as measles and adenoviral infection, and there is no single confirmatory test to specifically diagnose KD [1].

**Figure 2:**
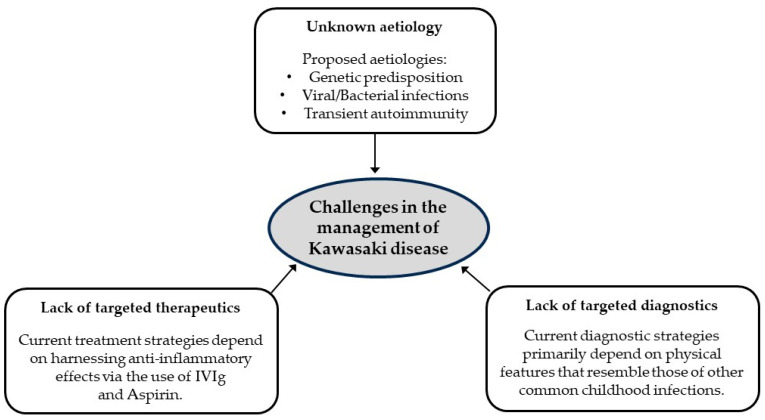
Knowledge gaps associated with Kawasaki disease.

Although current research findings robustly show that the fundamental basis of KD is vascular inflammation, its aetiology, targeted diagnostic tools and effective therapeutics are yet to be discovered (Figure 2). Hence, the development of a sensitive and specific test to diagnose KD in its early stages or incomplete manifestation remains one of the key pursuits in this field.

### 1.2. Liquid Biopsy

**Figure 3:**
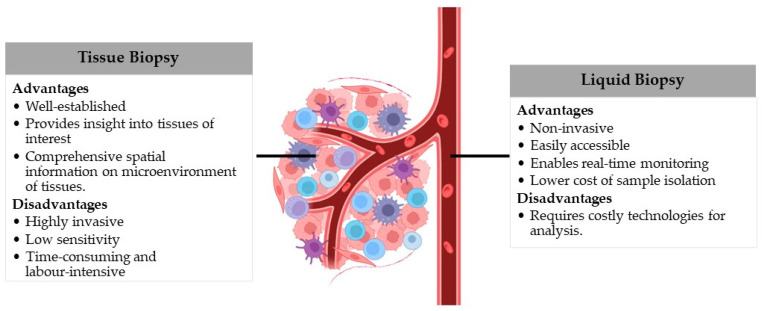
Evaluation of tissue and liquid biopsies.

The recent era has witnessed an explosion of interest in the use of accessible body fluids to identify and track disease. As an alternative to biopsy of solid tissue/organ, liquid biopsy involves sampling body fluids, usually for molecular components or cells released from the tissue/organ of interest [10]. Such body fluids include blood, plasma or serum, urine, saliva, cerebrospinal fluid and others (e.g., pleural effusion) [11,12]. Compared to tissue/organ biopsy, the liquid biopsy approach is advantageous because it is less invasive and less expensive, and it offers the convenience of serial biopsies for monitoring disease progression (Figure 3) [13,14,15,16,17]. Liquid biopsies can sample numerous molecular entities in the blood, including circulating tumour cells, circulating endothelial cells (CEC), circulating nucleic acids, such as cell-free nuclear DNA (cfDNA), cell-free mitochondrial DNA, circulating tumour DNA and cell-free RNA, as well as extracellular vesicles (often containing nucleic acid components).

Over the past few decades, there has been an exponential increase in research investigating liquid biopsies in a wide range of human diseases. Non-invasive prenatal testing to detect foetal cfDNA has been used to screen for the presence of chromosomal abnormalities, as well as to determine foetal sex. In oncology, there have been numerous applications of liquid biopsy [18,19,20], including the Epi proColon^®^ test, which analyses methylation patterns in cfDNA for population-wide colorectal cancer screening [21]. In the field of solid organ transplantation, the liquid biopsy approach has also been used for early detection of allograft rejection [22,23,24].

Here, we aim to review recent developments in the application of liquid biopsies to KD, summarising its role in the diagnosis, prognosis and monitoring of KD. We seek to provide scientists and clinicians with an overview of the current insights into the aetiology and pathogenesis of KD, for a better understanding of the basis for considering liquid biopsy as a diagnostic tool for KD.

## 2. Current Concepts in the Aetiology and Pathogenesis of Kawasaki Disease

### 2.1. Proposed Aetiologies of Kawasaki Disease

For the last five decades, researchers have been on an active quest, in search for the potential cause(s) and mechanisms underlying KD, to elucidate effective diagnostic tools and treatment targets. Current understanding suggests that the cause of KD is likely multifactorial, including genetic susceptibility, infectious triggers and immunological factors [25].

#### 2.1.1. Genetic Predisposition

The higher incidence among East Asians (especially Japanese) and the increased incidence in siblings of KD patients suggest a genetic predisposition to KD susceptibility [26,27,28,29,30,31]. Family linkage studies and genome-wide association studies, with subsequent validation studies, have implicated single-nucleotide polymorphisms (SNP) in six genes or gene regions: Fc fragment of IgG receptor IIa (*FCGR2A*), caspase-3 (*CASP3*), human leukocyte antigen class II, B-cell lymphoid kinase (*BLK*), inositol 1,4,5-trisphosphate kinase-C (*ITPKC*) and CD40.

*FCGR2A* and its SNP (rs1801274) are known to trigger an immune response via interactions with the IgG receptors on phagocytic cells (dendritic cells, macrophages, monocytes and neutrophils), which suggests a mechanistic basis for IVIg treatment [32,33,34]. Besides *FCGR2A*, other highly susceptible genes and their associated SNPs have been identified, but they are associated with other diseases as well (summarised in Table 1). There are also differences in genetic susceptibility across ethnicities. For example, *BLK* SNPs are more prevalent in Asian populations, while *FCGR2A* is prevalent in individuals of European descent [1,32,33,34,35]. Although SNPs for genes such as *ITPKC* and *CASP3* seem to be unique to KD based on a genome-wide linkage study, it warrants extensive research across various diseases to confirm its exclusivity. The SNPs of *ITPKC* and *CD40* trigger the activation of endothelial cells (EC), while the other SNPs (Table 1) are responsible for stimulating the activity of immune cells. These demonstrate the possible genetic influence on EC activation and immune cells in KD pathogenesis [32,33,34,35]. However, studies have not identified a specific gene to be primarily responsible for KD development or progression. Furthermore, genetic susceptibility as a sole aetiology seems unlikely, owing to the low recurrence rate, as one may expect a genetically predisposed patient to develop the disease more often in their lifetime.

On the note of genetic disposition, microRNA (miRNA), a non-coding RNA modulating the post-transcriptional modification of messenger RNA (mRNA), has gained interest in KD research recently [36]. MiRNA can trigger a pathogenic immune response, and its polymorphism highly correlates with autoimmune diseases [36]. Thus, scientists employed next-generation sequencing to identify miRNA indicating susceptibility to KD or CALs [37]. It has been shown that miRNA can not only induce a pathogenic response, but also a protective effect [36]. Thus, miRNA could serve as a useful diagnostic, prognostic or treatment strategy, as proposed by Xiong et al. [36] in a comprehensive review on miRNAs in KD. However, further research and clarification are necessary.

#### 2.1.2. Infectious Triggers

The clinical features (fever, rash, oral and conjunctival injection, lymphadenopathy), unique age distribution (between 6 months and 6 years of age), observation of community outbreaks and seasonal fluctuation of KD [1,2] mimic those of acute infections. Given that KD is accompanied by a systemic inflammatory overactivation, it is logical to propose the presence of superantigen(s) triggering KD. Although current findings suggest some viral and bacterial agents involved in KD pathogenesis, no single causative agent has been identified.

Bacterial aetiology is proposed based on similarity in clinical presentations, such as oral mucositis, cervical lymphadenitis and desquamation of hands and feet, which are related to diseases caused by staphylococci and haemolytic streptococci [1,6]. Since the gastrointestinal tract contains the largest lymphoid tissue, hosting a myriad of micro-organisms and biological agents, the mucosal membranes were investigated. The results suggested that certain antibiotic-resistant Gram-positive staphylococci and streptococci are involved in triggering KD [38].

On the other hand, respiratory viruses have been detected in nasopharyngeal aspirates in almost half of KD patients [39,40]. Viral aetiology is also postulated by the infiltration of immune cells, such as CD8^+^ T-lymphocytes, IgA plasma cells and macrophages, which occurs in any acute viral infections [6]. Electron microscopic studies on KD patient samples showed aggregates of RNA and viral protein in the ciliated bronchial epithelium, suggesting that an acute viral infection of the respiratory system could have progressed and resulted in KD, or that the causative agent enters the body through the respiratory system [6]. The viruses that are potentially involved are cytomegalovirus, adenovirus, rhinovirus, enterovirus and bocavirus [6]. On a separate note, since KD incidence is higher during certain seasons (i.e., winter and spring) and can occur in large numbers, it is theorised that a superantigen may be a trigger for KD. However, given the lower rate of incidence compared to seasonal flu [41], it is further theorised that the superantigen triggering KD may have other conditions, such as affecting only genetically susceptible persons. However, the low recurrence rate and rarity in adulthood cast doubt on this theory [6].

There are also findings to show that KD could be caused by a non-microbial antigen trigger. For example, transcriptomics of whole-blood samples from KD patients revealed that the majority of them are categorised under the non-viral and non-bacterial groups [42]. This suggests that KD patients presenting with bacterial or viral profiles may be experiencing a simultaneous infection by superantigen(s) and a separate trigger for KD [42]. Despite their possible role in KD development, there is no evidence that superantigens promote CALs [39], suggesting that they may not have a direct role in KD pathogenesis.

#### 2.1.3. Immunological Factors

The inflammatory basis of KD is not only supported by clinical manifestations but also by the underlying biology. This finding corroborates results from peripheral blood mononuclear single-cell RNA-sequencing, in which proinflammatory molecules (e.g., TNF, IL-1β, IFN-γ) are highly expressed, together with immunoglobulin receptors [43]. Inflammatory cells were also identified in the various layers of blood vessel via the careful study of arterial tissues from 41 KD patients [1]. This supports that KD is predominantly driven by vascular inflammation [44].

The immune system is designed to protect us from pathogens and their future infections in a systematic manner, via the innate and adaptive immune responses [45]. The innate immune response by a group of protein chemicals and phagocytic cells (e.g., macrophages and neutrophils) is the first line of defence against pathogens. The adaptive response provides a secondary, and more targeted, defence via T-lymphocytes, B-lymphocytes and antibodies [45]. However, some antibodies, known as autoantibodies, target self-antigens, triggering pathological responses [46]. Although the stimuli for autoantibody production are unclear, it has been observed that inflamed organs or tissues induce its production [46]. For example, autoimmune conditions such as systemic lupus erythematosus present several autoantibodies [46]. In addition, patients with vasculitis, such as KD, are reported to have anti-endothelial autoantibodies (AECAs) and their amounts in the sera are associated with the severity of the disease [47]. The specific antigenic targets of AECAs in KD are unknown, but they are suspected to induce EC activation and damage [47]. It is yet to be elucidated whether AECAs play a role in KD pathogenesis [47,48].

The aetiologies discussed above alluded to the involvement of immune activity on blood vessels as the basis of KD, which could result in endothelial injury. Thus, products of endothelial injury could potentially emerge as diagnostic tools for KD.

### 2.2. Pathogenesis of Kawasaki Disease

#### 2.2.1. Immune Response in Kawasaki Disease

The initial immunological reactions of KD consist of a trigger and an acute reactive phase. Several studies have shown that an undiscovered stimulus could trigger inflammatory cascades, with activation of both the innate and adaptive immune systems [49,50]. Although early studies suggested an immune response triggered by a superantigen, subsequent studies favoured a canonical response to a conventional antigen [6,51].

The innate immune system plays an important role during the acute phase of KD. Activation of the innate immune system needs to be tightly regulated; excessive activation can lead to systemic inflammation and tissue injury. The acute phase of KD is driven primarily by innate immune hyperactivation [52]. This is evident by the increase in the absolute neutrophil and monocyte counts in peripheral blood, and the observation of neutrophils and monocytes/macrophages being the major immune cell populations in coronary arterial lesions [53]. The higher expression of toll-like receptor 2 on the peripheral blood monocytes also indicates that innate immunity is a vital part in KD pathogenesis [54]. Furthermore, studies have shown increased serum IL-1β levels, as well as activation of the IL-1β signalling pathway in acute KD [55,56]. These findings point to the role of inflammasome activation in the immunopathogenesis of KD. Inflammasomes are large cytosolic multiprotein oligomers involved in sensing danger signals in innate immunity, with NLRP3 (nucleotide-binding domain and leucine-rich repeat pyrin domain containing 3) activation acting as a key component. This inflammasome serves as the intracellular machinery responsible for the production of important proinflammatory cytokines, such as interleukin-1β (IL-1β) and interleukin-18 (IL-18), thereby producing an inflammatory response. Indeed, the upregulation of NLRP3 mRNA [57] and increased proinflammatory mRNA regulating the NLRP3/caspase-1-dependent and caspase-4/5-dependent inflammasomes [51] were observed during the acute phase of KD, demonstrating the role of NLRP3 inflammasome regulation in KD immunobiology.

In addition to innate immunity, adaptive immune response has a significant role in the immunopathogenesis of KD. This is characterised by a decreased absolute T-cell count in peripheral blood, T-cell unresponsiveness to activation via the T-cell antigen receptor CD3 [58], downregulation of T-cell receptor and B-cell receptor signalling pathways [56,59,60,61], as well as decreased regulatory T- and B-cells during the acute phase of KD [62,63]. There is an accompanying Th17-related immune response and a strong inhibition of most T-cell and B-cell responses during the acute phase of KD [64]. The role of the adaptive immune system is further supported by the expansion of the regulatory T-cells after IVIg administration, accompanied by cessation of fever and clinical improvement [65].

#### 2.2.2. Vasculopathy Associated with Kawasaki Disease

##### Histopathological Aspects of Coronary Artery Lesions in Kawasaki Disease

The most significant complication related to KD is CALs, including coronary artery dilatation (ectasia) or coronary artery aneurysm, which could lead to coronary artery thrombosis and occlusion. It is proposed that KD-driven CALs develop due to inflammatory processes, such as infiltration by immune cells, inflammatory molecules, MMPs and TGF-β. These particles alter all three layers of the vessel tunicae – tunica intima (comprised of ECs and underlying basal lamina), tunica media (comprised of smooth muscle cells and connective tissues consisting of collagenous and elastic fibres), and tunica externa (comprised of collagenous fibres). These alterations could potentially develop into fusiform, saccular and ectatic aneurysms along the coronary arteries of the heart [66]. A model of KD vasculopathy proposed that three pathological processes stemming from inflammation, namely necrotising arteritis, subacute/chronic vasculitis and luminal myofibroblastic proliferation, are involved in CALs [67,68]. Necrotising arteritis is initiated by the infiltration of neutrophils into the endothelium, progressively necrotising the media and adventitia (tunica externa) layers, resulting in large aneurysms with a thin rim of adventitia. Subacute/chronic vasculitis is triggered by the infiltrations of leukocytes such as lymphocytes, plasma cells and eosinophils. It can affect all blood vessels, although preferentially medium-sized arteries, within two weeks post-fever. The injury progresses from the adventitial layer towards the lumen [67]. This is usually accompanied by luminal myofibroblastic proliferation, where the fibroblasts in the medial layer proliferate and potentially obstruct the arterial lumen [67]. These CALs can occur in 15–25% of untreated patients and could lead to severe cardiovascular complications, such as aneurysm rupture or acute myocardial infarction (AMI) from coronary thrombosis [1].

KD autopsy studies have demonstrated that KD vasculitis is characterised by granulomatous inflammation with monocytes/macrophage infiltrations, whereas fibrinoid necrosis rarely occurs. No immune complex depositions have been detected in KD vasculitis lesions. Thus, these pathological findings of KD are also distinct from those of immune complex-associated vasculitis [69,70]. The major cell populations present in human-autopsied KD specimens within 2 weeks after the disease onset are monocytes/macrophages and neutrophils [53].

##### The Role of Endothelial Cells in Immune Function and Its Activation in Kawasaki Disease

Vascular ECs have an extensive network, occupying more than 1000 m^2^ within the body, and are extremely important for maintaining homeostasis [71]. In addition to regulating blood flow, vascular tone and haemostasis, ECs play significant roles in regulating immune responses. In fact, ECs have been found to have functions similar to those of innate immune cells, carrying out functions that include cytokine secretion, phagocytic function, antigen presentation, pathogen-associated molecular patterns and damage-associated molecular pattern sensing, proinflammatory and immune-enhancing activities, as well as anti-inflammatory, immunosuppressive, migratory, heterogeneous, and plastic responses [72]. The endothelium serves as the interface between circulating inflammatory mediators and vascular media or adventitia, and it is therefore a prime target of inflammation during acute KD [73]. Following the aforementioned complex immune response, there is a significant overproduction of different cytokines, elevation of glycocalyx components (indicating glycocalyx damage) and endothelial activation in KD [74,75,76,77,78,79,80].

Inflammation can be both a cause and a consequence of increased oxidative stress. Proinflammatory cellular contents including the membrane phospholipids of damaged cells, known as damage-associated molecular patterns (DAMPs), are oxidised by reactive oxygen species (ROS). DAMPs, including oxidised phospholipids and low-density lipoproteins, activate ECs to further produce proinflammatory cytokines and ROS [81]. At the active sites of inflammation, inflammatory cells, vascular ECs and smooth muscle cells are all capable of releasing ROS, enzymes and chemical mediators, resulting in oxidative stress. Oxidative stress also stimulates the NF-κB pathway and expression of cytokines and chemokines to further enhance the inflammation. Thus, inflammation and oxidative stress closely interact and mutually amplify the effects of each other [82,83]. These processes induce the activation of endothelial NLRP3 inflammasome. In addition to producing an inflammatory response, activation of NLRP3 inflammasome also results in pyroptosis, a specific form of cell death that combines the characteristics of apoptotic and necrotic death pathways, of the ECs and monocytes [84].

##### Vascular Endothelial Dysfunction in Kawasaki-Disease-Associated Vasculitis

When ECs become dysfunctional, it could lead to serious consequences, such as vascular leakage, atherosclerosis and stroke [85]. Endothelial injury is indeed a hallmark of many human diseases [85], and especially KD, a systemic vasculitis. Vasculitis during acute KD is likely a consequence of increased microvascular permeability, upregulated expression of adhesion molecules on ECs, infiltration by inflammatory cells and endothelial dysfunction [86,87]. Vascular endothelial dysfunction involves the activation of apoptotic pathways, proliferation and migration and is central to KD-associated vasculitis. The molecular mechanisms of vascular EC injury and dysfunction in KD-associated vasculitis have been well reviewed elsewhere [36,88]. These include the role of non-coding RNAs (micro-RNAs, long non-coding RNAs), inflammatory cell activation, cytokine production, ROS accumulation and lipid oxidation.

In response to the increased cytokines and chemokines, circulating monocytes are recruited to activated ECs, where they subsequently differentiate into cardiac macrophages [89]. As a matter of fact, the major immune cell populations in the coronary arterial lesions are monocytes/macrophages and neutrophils [53]. These innate immune cells express high levels of effector molecules such as elastase and matrix metalloproteinases [90], thereby resulting in the destruction of the elastic lamina of the arterial wall. Neutrophils may contribute to vascular inflammation and vascular injury through the enhanced formation of neutrophil extracellular traps [91].

Vascular ECs are thought to be a source of myofibroblast-like cells, which have proinflammatory and profibrotic properties, through endothelial–mesenchymal transition (EndoMT). EndoMT describes the process by which ECs differentiate into mesenchymal cells, and EndoMT was found to be essential for cardiac valvular development and is involved in cardiovascular diseases such as myocardial infarction, cardiac fibrosis, endocardial fibroelastosis, valvular calcification, atherosclerosis and pulmonary hypertension [92]. Under various conditions, including inflammation and transforming growth factor β (TGF-β) signalling, ECs may undergo EndoMT, during which the expression of mesenchymal lineage markers is induced, and the EC lineage markers decrease. These myofibroblast-like cells, a set of spindle-shaped cells in the vascular media with a high expression of alpha-smooth muscle actin (α-SMA), participate in the recruitment of proinflammatory cells and induce arterial wall damage by secreting IL-17, MMPs and connective tissue growth factor. These myofibroblast-like cells are the presumed source for disordered collagen, which reduces the structural integrity of the media layer of arteries and contributes to aneurysm formation in KD [93]. KD autopsy studies with electron microscopy have confirmed the presence of myofibroblasts in the arterial wall, and these cells likely contribute to vascular fibrosis and remodelling [68].

Since endothelial injury is consistently present in KD, it will be useful to explore the direct markers of endothelial injury as diagnostic tools for KD. Although multiple non-endothelial specific protein biomarkers such as VWF/antigen and C-reactive protein (CRP) have been investigated for KD diagnostic application, many lacked specificity for KD, and a few (e.g., VWF/antigen) need to be thoroughly validated prior to clinical applications [94,95]. Thus, direct markers of endothelial injury, such as circulating endothelial cells (CECs), endothelial microparticles (EMPs) and vascular endothelial cell-free DNA (EC-cfDNA), could potentially serve as straightforward and reliable diagnostic markers of KD.

## 3. Application of Liquid Biopsy in Vasculopathy

Since ECs are in direct contact with circulating inflammatory mediators, it is expected that the products of injured vascular ECs will be released into the bloodstream. Such products include CECs, EMPs, EC-cfDNA, microRNA and endothelial-specific proteins or compounds. Hence, liquid biopsy seems promising for clinical or research applications relating to vascular diseases [17]. Here, we review the potential of CECs, EMPs and EC-cfDNA obtained via blood collection, as diagnostic tools for KD (Table 2).

### 3.1. Circulating Endothelial Cells

**Figure 4:**
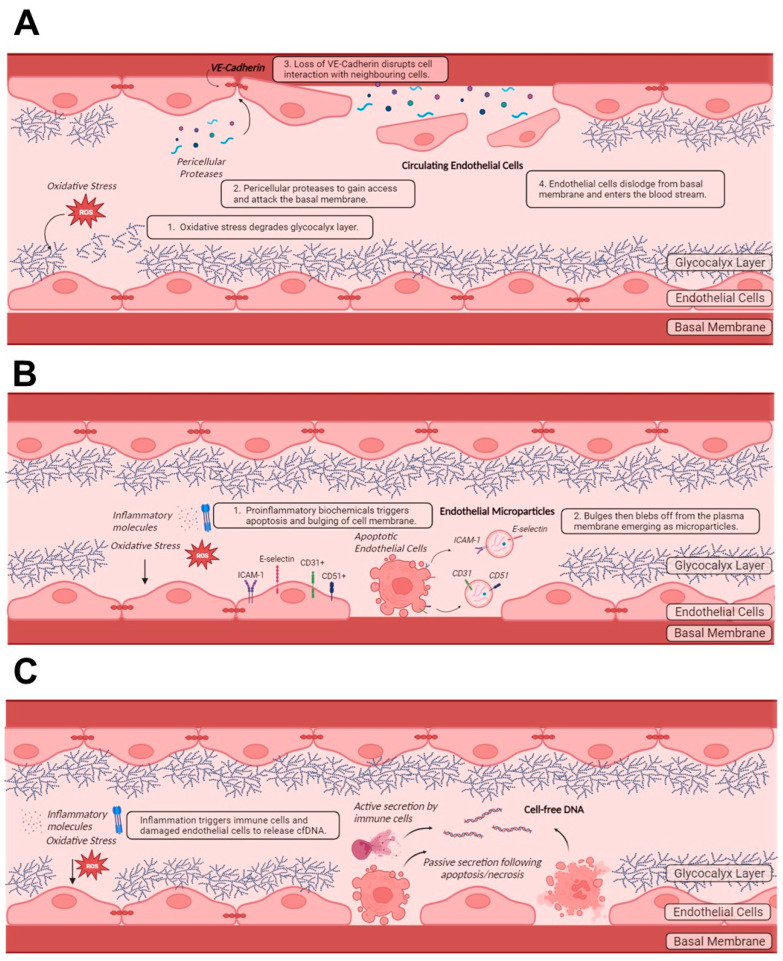
Mechanisms underlying the release of endothelial damage products into peripheral blood circulation. Release of (**A**) endothelial cells, (**B**) endothelial microparticles and (**C**) cell-free DNA into the peripheral blood stream.

When oxidative stress, due to infections or inflammation, is induced on the endothelium, the glycocalyx layer and nitric oxide balance become disrupted (Figure 4A). This increases the permeability of the endothelium [85,96,97]. These processes describe endothelial injury. This allows for immune molecules or pericellular proteases to gain access and attack the basal membrane, disrupting the adhesion of ECs to the extracellular matrix and to the neighbouring cells through loss of vascular endothelial (VE)–cadherin-mediated action (Figure 4A). This eventually results in the dislodgement of ECs from the basal membrane to enter the blood circulation, becoming CECs [98,99]. CECs could also emerge owing to mechanical injury and drug-induced desquamation [100]. CECs are nucleated, with a size of approximately 10–50 µm and a morphology similar to mature ECs [71,100]. They could adopt various phenotypes, such as activated, apoptotic or necrotic cellular states [100], depending on their disease states. It has been proposed that the surface markers differ based on their origin and disease state, although this has not been established. Using flow cytometry, a multicolour panel is used to detect CECs, irrespective of their cellular states. The established set of panels include a combination of CD146^+^, CD45^−^, CD31^+^, CD133^−^ and Hoechst 33342, which excludes cells with hematopoietic (CD45^−^) and progenitor markers (CD133^−^) and includes only matured nucleated cells (Hoechst 33342) with endothelial markers (CD146^+^ and CD31^+^) [100]. Besides flow cytometry, immunomagnetic capture can also be used to detect CECs [101].

The utility of CECs as diagnostic and prognostic markers has been demonstrated. The number of CECs generally increases with cardiovascular risk factors and diseases [99]. For example, the accuracy in diagnosing unstable angina was significantly improved when both cardiac troponin and CECs were used as diagnostic markers, as the increase in CECs occurs sooner and is independent of the changes in troponin [99,102]. In addition, the enumeration of CECs at the onset of acute coronary syndrome appeared to be promising in predicting long-term outcomes, such as major adverse cardiovascular events or even death [103]. CEC counts could also be used to assess endothelial function as they are inversely correlated with flow-mediated dilatation [104]. However, owing to their lack of specificity towards any disease, CECs can only be part of a multi-model diagnostic strategy. Nevertheless, their clinical application in KD has yet to be established.

#### Circulating Endothelial Cells as Diagnostic Tools for Kawasaki Disease

A search of PubMed, Scopus and ScienceDirect, with the terms (Kawasaki) AND (“circulating endothelial cells”), resulted in 74 publications between 2003 and 2023. Only 10 publications, all of which were original research articles, reported on CECs in the context of KD [101,105,106,107,108,109,110,111,112,113]. These articles were published from 2003 onwards and their research findings are summarized in Table 3. 

Nakatani et al. and Fu et al. have reported that CECs are generally higher in the acute (~3–10 days of fever) and subacute (~10–21 days) phases of KD, when compared to the convalescent phase (22–60 days) and healthy controls. They also reported that patients with coronary artery lesions (CALs) have significantly higher CECs in the acute and subacute phases, as compared to those without CALs [110,113]. This is expected, as CALs occur as a result of inflammatory cell infiltration, vascular oedema and eventual loss of structural integrity, encouraging the displacement of ECs into the bloodstream [101]. Since the CEC amounts were reported in different units, there is no basis of comparison for the data from both studies and only data trends could be compared. For example, Nakatani et al. and others reported CEC counts with respect to per millilitre of blood (CECs/mL) [105,112,113], while Fu et al. and some others reported the number of CECs with respect to the number of mononuclear cells [107,110].

The possibility of using CECs as a marker of endothelial injury was assessed in inflammatory diseases, such as KD, multisystem inflammatory syndrome in children (MIS-C) and COVID-19 infection [101]. CECs were higher in KD, as compared to MIS-C, in both acute (≤10 days of fever) and subacute (11–20 days after fever) phases, suggesting that KD can be differentiated from MIS-C based on CEC counts [101]. However, the dispersion of data for CECs in acute COVID-19 was too large to be conclusive, and there was no correlation between CEC counts and clinical characteristics of children diagnosed with COVID-19 [101]. In accordance with CEC numbers, the coronary artery (CA) dimensions were larger in KD compared to the non-KD febrile or inflammatory diseases. Moreover, in KD, the regression of CALs was limited compared to MIS-C, suggesting that KD injures the endothelium in a more aggressive manner. Collectively, the significant difference in the CEC counts and CA dimensions of both diseases implies there are different pathways leading to the respective diseases [101], and that it is more favourable to use CECs for the diagnosis of KD. The next few paragraphs detail the mechanisms known to trigger the elevation of CECs.

The role of S100 family proteins, a DAMP, in releasing ECs from the basal membrane under the conditions of KD has been well investigated. The S100 family heterodimer myeloid-related protein (MRP)-8 and -14 are secreted by activated granulocytes and monocytes under inflammatory conditions and are found in infiltrating macrophages and neutrophils [114]. MRP-8 and -14 proteins are known to bind to endothelial glycocalyx, potentially triggering an adverse response on the ECs [111]. The S100A12 protein, a member of the S100 family, binds to the receptor for advanced glycation end-products (RAGE) on the endothelium, inducing a NF-κB-dependent activation, and hence triggering the release of proinflammatory cytokines such as TNF and IL-1β [109,115]. Since an inflammatory response could trigger EC injury, it is postulated that the S100 family proteins play a role in the generation of CECs in KD patients. Supporting this, Hirono et al. and Wang et al. concluded that the levels of MRP-8/MRP-14 proteins in serum and MRP-8/MRP-14-positive CECs may be useful markers of KD disease severity [108,111]. Fu et al. reported that the expressions of S100A12 on the surface of CECs increases significantly in KD patients and remains elevated for an extended period in patients with CALs [110].

Gong et al. reported that the expression activity of RAGE on CECs increases significantly in KD patients and progressively increases in patients with CAL [109]. C-reactive proteins (CRPs), which are elevated in children with KD, are known to enhance RAGE expressions on ECs and promote CECs [106,116]. Zhou et al. demonstrated that RAGE is necessary for CRPs to trigger the release of ECs into the circulation, although this finding is not specific to KD [106].

Nitric oxide (NO) is crucial for vasoprotection [117]. However, NO levels need to be regulated, as an excess or deficiency could cause endothelial dysfunction [117]. NO is synthesized by nitric oxide synthase (NOS) isoforms, endothelial NOS and inducible NOS (iNOS). In general, the iNOS expressions and CECs are higher in KD patients with CALs [112]. In addition, iNOS was detected in the ECs from coronary artery aneurysms on histology [112].

The research findings on the aforementioned proteins and inorganic compounds show that inflammatory molecules, which are postulated to facilitate KD vasculitis, are closely associated with the production of CECs, and potentially the development of CALs. This demonstrates the relevance and reliability of CECs as a diagnostic marker of KD.

Both Shah et al. and Mostafavi et al. concluded the presence of long-term vascular damage based on the amounts of CECs in patients’ blood at ten years post-KD [105,107]. Shah et al. also conducted an extensive analysis on various other markers, such as EMP, soluble cell-adhesion molecule cytokines, cardiovascular risk factors, pulse-wave velocity and carotid intima media thickness. Besides CECs, CD105+EMP, soluble vascular cell adhesion molecule-1 and soluble intercellular adhesion molecule-1 were significantly higher in the KD group compared to healthy controls. It is worth noting that about 45% of the study population who had coronary aneurysms during KD had persistent dilatations of the coronary artery at the time of the study. Patients with persistent coronary aneurysm had the highest CECs, but even those with regressed coronary artery aneurysm had higher CECs than healthy controls. [105] The possible application of CD105+EMP will be discussed below. The study comprehensively featured the suitability of using CECs for long-term surveillance of vascular health post-KD.

These studies collectively show that CECs have immense potential as diagnostic tools and prognostics for KD. The ongoing efforts and future research aimed at their implementation in the clinical settings are discussed in Section 4.1.

### 3.2. Endothelial Microparticles

Microparticles are plasma membrane-shed vesicles from activated or apoptotic cells. The imbalance of transmembrane enzymes results in the breakdown of cytoskeletal fibres [118], causing a bulge to be formed on the plasma membrane. This bulge then blebs off from the plasma membrane, taking part of the cytoplasm with it, emerging as microparticles. The process of blebbing is facilitated by proinflammatory biochemicals such as TNF-α, ROS and cytokines [119]. The biomolecules on microparticles provide information on the type of cell from which the microparticles originated [120].

It has been reported that there are 10^3^ and 10^5^ EMPs per ml of plasma [121]. Since EMPs are derived from ECs (Figure 4B), these vesicles present typical endothelial proteins as surface antigens (e.g., CD31, CD51, CD54, CD62, CD104, CD105, CD106, CD144, CD146), depending on the state of the ECs from which they emerged. These antigens distinguish the EMPs from microparticles of other cellular origin [120]. EMPs also carry nucleic material, such as DNA and RNA, which can be examined further to identify the organ from which these EMPs originated [120,122]. These may be employed in the assessment of endothelial damage in specific organs. Enzyme-linked immunosorbent assay (ELISA) and flow cytometry principles can be used to identify the EMPs [123]. However, since the size of the EMPs is similar to that of neutrophils, platelets and cell fragments, the sensitivity and accuracy of the flow cytometer and hence the data must be verified [71].

Lugo-Gavidia et al. demonstrated a positive correlation between cardiovascular diseases and EMPs across various publications [124]. Mizrachi et al. has shown that EMPs (CD31^+^ and CD51^+^) were significantly elevated in patients with coronary artery disease (CAD) compared to controls [125]. An increase in EMP (CD31^+^) was associated with an impairment in endothelial-dependent vasodilation in patients with CAD [126]. These studies displayed the capability of EMPs as cardiovascular diagnostic tools.

EMPs could also present proteins such as phosphatidylserines on their surface, demonstrating procoagulant and proinflammatory characteristics [119]. Although EMPs emerge from damaged or dying cells and are reputed to contribute to inflammation and the progression of vascular diseases, recent findings suggest that they could facilitate favourable processes, such as cell survival, anti-inflammation, anti-coagulation, and even induce endothelial regeneration [120]. Thus, these multifaceted roles of EMPs must be clarified while exploring their clinical utility. In addition, it has been reported that EMPs tend to be cleared from the circulation within an hour after an event of cardiac stress [127]. Should the rate of clearance be similar in the context of vascular diseases, the application of EMPs as diagnostic tools will be limited [121]. However, there is a lack of information on this, and it should be explored further to draw a comprehensive conclusion on the role of EMPs as diagnostic tools.

#### Endothelial Microparticles as Diagnostic Tools for Kawasaki Disease

ECs are the main contributor of microparticles, and they are significantly elevated in KD patients [128,129]. The shedding of the EMPs from the plasma membranes was observed in a rabbit model of coronary artery vasculitis [130]. The potential of EMPs as diagnostic markers of KD is supported by their correlation with vascular dysfunction, determined by brachial artery flow-mediated dilation [131].

A search on PubMed with the terms ‘Kawasaki disease and endothelial microparticle’ and ‘“Kawasaki disease” and “endothelial microparticle”’ resulted in a total of 10 publications (between 2004 and 2023), of which one publication was excluded, as the focus was on platelet microparticles and not EMPs [132]. Four of the studies demonstrated the potential diagnostic tools for KD by reporting the associations between EMPs and KD [105,128,131,133]. Four other studies described the direct or indirect contributions of EMPs towards KD [118,129,134,135]. The final publication, which was mentioned earlier, had structurally captured the development of EMPs in coronary arteritis using a KD rabbit model [130]. These are summarised in Table 4.

Tan et al. reported that EMPs (CD31^+^ and CD146^+^) were increased in KD patients by 1.6-fold and 2.4-fold when compared to febrile and healthy controls, respectively, before IVIg treatment [133]. These results were corroborated by the work of Nakaoka et al., who included only CD144^+^ EMPs. They reported a statistically significant increase in KD patients prior to IVIg treatment, when compared to febrile and healthy controls, respectively [129]. Ding et al. showed that the EMPs (CD144^+^, CD62E^+^ and CD105^+^) were elevated during acute, subacute and convalescent phases (time frame not reported) of KD when compared to healthy controls [131]. Similar to CECs, the EMPs peaked during the subacute phase [110,113,131]. However, contrary to the aforementioned studies, the EMPs in the KD patients were not statistically different compared to the febrile controls across the three phases of KD [131]. The inconsistency in the trend presents a need for a systematic approach, that is, using a fixed surface antigen as reference biomarker to evaluate the reliability of EMPs as diagnostic tools. A robust protocol will be necessary to standardise detection techniques and reporting standards which are now highly inconsistent (summarised in Table 4).

Shah et al. demonstrated the extent of endothelial damage approximately 8.3 years after the occurrence of KD [105]. The EMPs were enumerated by targeting various surface antigens (e.g., CD105+, E-selectin^+^, ICAM-1^+^, VCAM-1^+^, CD144+, CD31+). However, only the CD105^+^ EMP was significantly elevated in the KD survivors who had had coronary aneurysms when compared to healthy controls [105], and there were no statistical differences when the KD survivors with no coronary aneurysms were compared to healthy controls instead. This showed that CD105^+^ EMPs may be useful as a surveillance marker to assess long-term vascular health in KD survivors, especially those who had KD-related coronary complications [105].

CD105, also known as Endoglin, is a transmembrane glycoprotein expressed on ECs, which functions as a co-receptor for the TGF-β family and as an angiogenesis marker [136]. Interestingly, a significant increase in vascular endothelial growth factor (VEGF) was observed in KD patients with coronary aneurysms when compared to healthy controls. The significant elevation in both CD105^+^ EMPs and VEGF shows that the vascular homeostasis had been disrupted for an extended period and vascular repair was still ongoing years after KD [105], highlighting the need for long-term cardiovascular surveillance in KD patients with coronary complications. Yet, before asserting any claims, it is imperative to elucidate the role of CD105+ EMPs, considering their potential dual function as markers of both vascular damage and recovery. On the other hand, when CECs were enumerated from the same population, CECs were significantly elevated in the KD survivors, and even more so in the KD survivors who had had coronary aneurysms [105]. This finding is important, as it suggests that CECs may be more appropriate, in terms of sensitivity and role, than EMPs as surveillance or even diagnostic markers for KD. Overall, there are conflicting data on the utility of EMPs as diagnostic markers. This warrants further investigation, which is discussed in Section 4.2.

### 3.3. Vascular Endothelial Cell Specific Cell-Free DNA

cfDNA consists of short fragments of DNA released from dying cells (passive release) or specialised cells (active release) into the circulation (Figure 4C) [137,138]. Since its discovery, about 70 years ago, in human plasma, it has been an area of research interest for clinical applications owing to its non-invasive nature [138]. The characteristics of cfDNA could vary in size, DNA methylation and repeating sequences [139]. These characteristics differ based on the type of disease, and hence they may be useful in the detection of specific diseases. It has also been reported that cfDNA could trigger cytokine release and contribute to inflammation in a positive feedback manner [138]. Studies have shown that cfDNA is elevated under pathological conditions such as systemic lupus erythematosus, cancer and myocardial infarction [138]. However, a limitation of cfDNA is that it is present is small quantity. Although the DNA methylation pattern has been successfully detected in 1 mL of plasma (~3 mL of blood), it also depends on the extent of cellular damage that occurs in the disease state [140]. Hence, in certain pathologies, a higher volume of blood may be necessary to achieve a detectable concentration of cfDNA. Furthermore, although it is known that cfDNA is cleared via renal excretion, liver and spleen metabolism and is suggested to circulate between 16 minutes and 2.5 hours, there are limited information on the rate of cfDNA clearance, which limits the utility of cfDNA as a diagnostic tool [141,142]. Should these limitations be overcome, the access to cfDNA would tremendously improve and emerge as a powerful non-invasive diagnostic tool.

**Figure 5:**
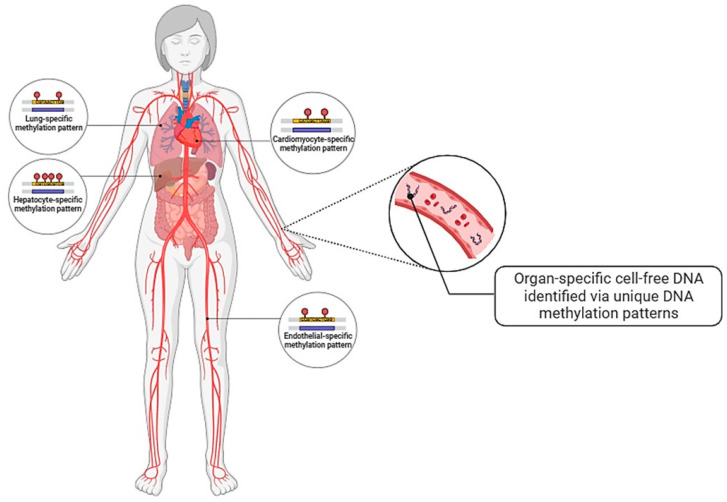
Application of cell-free DNA (cfDNA) in liquid biopsy. The non-genetic features of cfDNA, including CpG methylation patterns, can allow for identification of their tissue and cellular origin.

Recently, tissue-specific cfDNA has garnered interest in diagnostic research (Figure 5). Utilising DNA methylome tools such as comparative methylome analysis, unmethylated DNA sequences that were unique to cardiomyocytes were identified [140]. This allowed for cardiomyocyte-specific cfDNA to be detected and used as marker of cardiomyocyte death. Zemmour et al. has demonstrated that patients with acute ST-elevation myocardial infarction had significantly elevated cardiomyocyte-specific cfDNA, which corresponds to the elevation of cardiac troponin levels, whereas hardly any cardiomyocyte-specific cfDNA signals were detected in the plasma of healthy individuals [140]. However, cardiomyocyte-specific cfDNA seems to be slightly inferior to the sensitivity of troponin-T [140]. To overcome such limitation, multiplex detection of cardiac-specific unmethylation sequences can be considered. Nonetheless, although it is not tissue-specific, there are successful clinical applications of cfDNA, such as Allosure^®^, where SNPs of donor-derived cfDNA are quantified in renal transplant recipients to assess the possibility of allograft rejection [143].

Moss et al. has demonstrated the possibility of establishing an atlas of tissue- or organ-specific methylation patterns by comprehensively describing a methodology to achieve it [144]. Although such a database has not been established yet, it is a promising area of research to achieve quick, yet accurate diagnoses. For example, with known organ-specific DNA methylation patterns, cfDNA can be conveniently extracted from blood plasma, processed (i.e., bisulphite conversion) and quantified for the organ-specific methylated sequences [145]. This allows us to assess the damage to specific organs due to the pathologic conditions.

Interestingly, in the plasma of healthy participants, 10% of the cfDNA originates from vascular ECs, being the highest non-haematopoietic contributor of cfDNA [144]. This substantial amount has enabled Peretz at al. to identify robust vascular EC-specific cell-free DNA (EC-cfDNA) sequences. They have also stepped up a notch by identifying organ-specific EC-cfDNA sequences, such as lung-specific EC-cfDNA [145]. The data have shown that the quantification of lung specific EC-cfDNA is higher in lung-related pathologies (e.g., chronic obstructive pulmonary disease) compared to non-lung pathologies such as myocardial infarction, demonstrating the specificity of DNA methylation sequences.

A potential challenge is contamination of the EC-cfDNA signal by erythrocyte progenitor cells (EPCs), as they contribute to 30% of the cfDNA population and are heavily demethylated [144,145]. However, it has been shown that only 10% of EPC-cfDNA has similar methylation patterns to those of EC-cfDNA, and thus the contamination will appear as noise and is unlikely to interfere with the signal from EC-cfDNA [145]. Thus, although it seems that EC-cfDNA is promising as a diagnostic tool for KD, it needs to be thoroughly validated prior to clinical applications.

#### EC-cfDNA in Kawasaki Disease

Since the quantity of EC-cfDNA reflects the extent of EC damage, it is intuitive that it could play a role as diagnostic tool for KD. However, this application is in its early days, and there are hardly any findings reported in the context of KD. A search on PubMed with the terms ‘(“cell free DNA”) AND (“Kawasaki disease”)’ and ‘(“cell-free DNA”) AND (“Kawasaki disease”)’, provided only one publication by Yoshida et al. (2020), who investigated the association between neutrophil extracellular traps and KD by treating HUVECs with neutrophils from KD patients in vitro. cfDNA titre was used as a marker of cellular damage [91]. Thus far, there are no reports on the use of cfDNA or EC-cfDNA as a diagnostic tool for KD. Hence, at present, it is not possible to compare and evaluate the utility of EC-cfDNA against other markers of endothelial injury for KD diagnosis.

## 4. Future Directions

### 4.1. Circulating Endothelial Cells

Since it has been established that the number of CECs is elevated in KD patients and even more so in patients with coronary complications, it will be useful to establish CEC threshold counts as part of a multi-marker model to diagnose KD and for early detection of coronary abnormalities. To establish suitable thresholds, a multi-centre enumeration of CECs must be conducted for a large cohort of KD patients of diverse demographics to discern KD from other febrile illnesses. These threshold values must be validated against disease cohorts before using them in clinical settings. It has been postulated that it takes about seven days for CECs to return to baseline after angioplasty, although a comprehensive investigation has not been performed [146]. Should this rate be applicable to all cardiovascular diseases, the delayed clearance would provide a longer timeframe to use CECs as diagnostic tools. However, a limitation of CECs is their minute amount in peripheral blood circulation. For example, an average of 12.9 CECs were detected per millilitre of blood in healthy patients, although the numbers increase at onset of cardiovascular diseases [147]. Thus, a sensitive and robust technique is required to report reliable data, which is necessary to implement CECs as multi-diagnostic markers. In efforts to address this, Lanuti et al. conducted a multicentre investigation to standardise a robust flow cytometry approach [147]. Such standardised protocols will help to overcome the variability in reporting standards, as shown in Table 3. These would allow for establishing a reliable threshold for KD diagnosis, the prognosis of KD-related vascular complications and long-term cardiovascular surveillance in KD survivors. The workflow of flow cytometry (i.e., from isolating blood mononuclear cells to cell staining) takes about 2.5 h. While this duration does not significantly delay the ideal treatment window (i.e., >5 days of persistent fever) [148], reducing the workflow time would still be beneficial.

In terms of biomedical research applications, it is intuitive to culture the isolated CECs for in vitro investigations. However, CECs have poor proliferative potential. Nevertheless, CECs can be used for molecular profiling to gain mechanistic insights underpinning disease progression. Pathogens could be identified in CECs, making them useful for elucidating disease-causing agents [71].

### 4.2. Endothelial Microparticles

The use of various endothelial-specific surface antigens has brought about conflicting opinions on their role as diagnostic markers. Hence, thorough and systematic investigation could harness the usefulness of the individual surface markers for diagnosis, as specific surface markers could inform the function and disease status of ECs which are useful for uncovering the mechanisms underlying KD [118,129,135]. For instance, CD62E^+^ EMP reveals that the ECs are in the activated state [131]. Other research has shown that proteomics on EMPs from diseased states could reveal the mechanisms underlying disease progression [149,150].

Furthermore, establishing a baseline physiological level proves challenging due to varied reporting standards across studies [121,151]. On a technical front, robust protocols must be established for the detection of EMPs, despite their rapid clearance. These efforts are crucial for identifying suitable surface antigens for diagnosing Kawasaki disease (KD) and prognosticating associated coronary complications effectively.

### 4.3. Vascular Endothelial Cell Specific Cell-Free DNA

There is a lack of research on EC-cfDNA, especially in the context of KD, owing to its novelty. Thus, the first step is to validate the suitability of EC-cfDNA sequences by evaluating the sensitivity in assessing KD progression. Then, robust protocols must be established for the detection of EC-cfDNA despite their rapid clearance prior to establishing threshold values for diagnostic tools. From the biomedical research perspective, the type of release of cfDNA could be studied to uncover the mechanisms underlying disease progression. Although the majority of cfDNA is released via passive action such as apoptosis, cfDNA released actively, such as cell secretion via exosomes, can be differentiated by its genomic size [142].

## 5. Conclusions and Perspectives

It is a challenge to diagnose KD due to the lack of a specific diagnostic test. Delay in diagnosis and treatment predisposes the patient to the development of coronary complications, which is the leading cause of acquired heart disease in children [1]. Thus, this review reiterates the research gaps associated with KD and evaluates the potential of endothelial damage markers obtained from blood sampling (liquid biopsy) as diagnostic tools for KD. Although strong correlations have been reported between CEC counts and disease severity, there is a need to standardise reporting standards and establish threshold cell counts to facilitate clinical application. The delay in the clearance of CECs from the circulation could enhance the signal-to-noise ratio, making it an attractive diagnostic tool. With these establishments, CECs could even be used for the long-term surveillance of cardiovascular health post-KD.

While there is evidence of EMPs indicating endothelial damage, there is variability in the role of the different surface antigens, which needs clarification. In addition, it has been demonstrated that EMPs are cleared from the circulation within a couple of hours, suggesting that EMPs may only be useful within a narrow window of time.

EC-cfDNA has been identified very recently and its potential for diagnosing KD has not been demonstrated yet. Similar to EMPs, it has a rapid clearance rate, and hence it must be strategically employed as a diagnostic tool.

Currently, echocardiography is performed to identify complications in the proximal artery segments. However, subtle coronary dilations in the early stages of the disease are commonly undetected on echocardiogram, which could eventually develop into CALs by the sub-acute phase. Thus, it will also be useful to explore the capability of the aforementioned endothelial damage markers in prognosticating coronary complications at early stages in KD patients.

In conclusion, the use of circulating endothelial damage products as diagnostic tools for KD is a fresh, yet promising idea that warrants further investigation and verification before its implementation in clinical settings.

## Figures and Tables

**Table 1:** Examples of genes susceptibility to Kawasaki disease.

	Susceptibility Gene	Associated SNPs	Type of Study	Association with KD	Association of SNP with Other Pathologies	Association of Genes with Other Pathologies	Predisposed Ethnicity	Ref.
Family-based studies	Inositol 1,4,5-trisphosphate 3-kinase C (*ITPKC*)	rs28493229	Case-control association studies	*ITPKC* negatively regulates the signalling cascade triggered by inositol 1,4,5-trisphosphate (IP3) and the nuclear factor of activated T-cells (NFATs), which activate inflammatory and vascular ECs. However, its SNPs reduce expression of *ITPKC* mRNA.	None.	Hydrops ff gallbladder Bacterial conjunctivitis	Japanese, Taiwanese, Koreans, Chinese, Euro-American	[32]
	Caspase-3 (*CASP3*)	*rs113420705* (*formerly rs72689236*)	Case-control association studies	*CASP3* also inhibits the activity of IP3 and NFATs and mediates cellular apoptosis. However, its SNP reduces *CASP3* expression, limiting cellular apoptosis and sustaining potency of immune cells.	None.	Oropharynx cancer Retinal ischemia Monocytic leukaemia	Japanese, Taiwanese, Koreans, Chinese, Euro-American	[32]
Population-based studies	Fc gamma receptor IIa (*FCGR2A*)	rs1801274	Genome-wide association studies (GWAS)	*FCGR2A* activates and triggers a signal when conjugated with immune cells. SNP increases affinity to IgG receptors, enhancing phagocytic cell activation. This provides a basis, although not established, for IVIg treatment in KD.	Lupus nephritis Malaria Pseudomonas aeruginosa (cystic fibrosis)	Cystic fibrosis Systemic lupus erythematosus	European descent, Taiwanese, Koreans, Han Chinese	[32,33,34]
	B lymphoid tyrosine kinase (*BLK*)	rs2736340	GWAS	The SNP reduces *BLK* mRNA expression in B-cells, which may alter their activity to trigger the pathogenesis of KD.	None.	Rheumatoid arthritis Systemic lupus erythematosus	Japanese, Taiwanese, Koreans	[32,33,34,35]
	*CD40*	rs1883832	GWAS	SNP increases CD40 expression on B-cells leading to enhanced B-cell activity, which is suggested to be commonly involved in the pathogenesis of KD and other adult autoimmune diseases. It is known to enhance activation of inflammatory and vascular ECs.	Hyper-IgM syndrome type 3	Rheumatoid arthritis Systemic lupus erythematosus Autosomal recessive hyper-IgM immunodeficiency type 3.	Japanese, Taiwanese, Koreans	[32]

**Table 2:** Evaluation of endothelial damage products as diagnostic tools for Kawasaki disease.

Potential Diagnostic Tools	Advantages	Disadvantages
Circulating endothelial cells	Can be extracted from peripheral blood. Existing studies demonstrate their potential as diagnostic tools Surface antigens describe EC status.	Exist in low amounts in blood.
Endothelial microparticles	Can be extracted from peripheral blood. Surface antigens describe EC status.	Ambiguity regarding their role as endothelial damage markers. Ambiguity regarding their sensitivity as diagnostic tools. Fast clearance from circulation.
Endothelial-specific cell-free DNA	Can be extracted from peripheral blood. Has potential to identify organ-specific ECs.	Lack of studies, especially in the setting of KD. Fast clearance from circulation.

**Table 3:** Summary of studies on circulating endothelial cells and Kawasaki disease.

Literature	Type of Participants	Age (in Years, Median/ Range)	Female, n, %	Acute Phase	No. of CECs (Acute)	Sub-Acute Phase	No. of CECs (Sub-Acute)	Convalescent Phase	No. of CECs (Convalescent)	Long-Term Outcomes	Healthy Controls	CEC Detection Method	Biomarkers for Detection
Fabi et al. (2022) [101]	Active	1.8 (0.6–2.4 (IQR))	6 (66.7%)	1st–10th day of fever	16.3 (13.6–48.8)/mL of blood	11th–20th day after fever	45.8 (18.5–131.0)/mL of blood	-	-	-	-	Immunomagnetic capture	CD146
Shah et al. (2015) [105]	Survivors	11.9 (4.3–32.2) Age at diagnosis: 4.9 (0.18–11.3)	45 (49%)	-	-	-	-	-	-	8.3 years post-KD CECs: 24 cells/mL	n = 51 CECs: 49 cell/mL	Immunomagnetic capture	CD146
Zhou et al. (2015) [106]	In vitro model	-	-	-	-	-	-	-	-	-		Flow cytometry	CD146+, CD105+, CD45−, CD3+
Mostafavi et al. (2014) [107]	Survivors	6.6 (4.8–9.6)	8 (61.5%)	-	-	-	-	-	-	4–19 years post-KD CECs: 12 cells	n = 13 CECs: 2.38 cells	Flow cytometry	CD45−, CD34+, CD146+
Wang et al. (2014) [108]	Active	0.1–5	17 (41.4%)	During hospitalisation	392/mL of blood (unique formula was used)	-	-	-	-	-	-	Flow cytometry	CD45−, CD146+
Gong et al. (2012) [109]	Active	0.25–12.7	37 (41.6%)	4–10 day of disease	absolute count of CEC not reported	11–21 day of disease	absolute count of CEC not reported	22–60 days of disease	absolute count of CEC not reported	-	n = 38 absolute count of CEC not reported	Flow cytometry	CD45−, CD146+
Fu et al. (2010) [110]	Active	0.25–11	16 (38.1%)	4–10 day of disease	absolute count of CEC not reported	11–21 day of disease	absolute count of CEC not reported	22–60 days of disease	absolute count of CEC not reported	-	n = 60 absolute count of CEC not reported	Flow cytometry	CD45−, CD146+
Hirono et al. (2006) [111]	Active	0.16–7.3	21 (34.4%)	At diagnosis	2.5 cells/mL	2 weeks from onset	20.7 cells/mL	-	-	-	n = 33 1.0 cells/mL	Buffy-coat smears	P1H12 antibody
Yu et al. (2004) [112]	Active	0.3–7.25	29 (52.7%)	Before IVIg After IVIg	0.7 cells/mL 4.9 cells/mL	2 weeks from onset	24.4 cells/mL	4 weeks from onset	3.7 cells/mL	-	n = 15	Buffy-coat smears	P1H12 antibody
Nakatani et al. (2003) [113]	Active	0.67–6	5 (25%)	Before IVIg therapy on days 3–7	16.4 cells/mL	After IVIg therapy on days 9–16	21 cells/mL	days 22–37	9 cells/mL	-	n = 10 < 6 cells/mL	Immunomagnetic capture	P1H12 antibody

**Table 4:** Summary of studies on endothelial microparticles and Kawasaki disease.

Literature	Type of Participants	Age (Median Years (Range))	Female (%)	Acute Phase	No. of EMPs (Acute)	Sub-Acute Phase	No. of EMPs (Sub-Acute)	Convalescent Phase	No. of EMPs (Convalescent)	Long-Term Outcomes	Healthy Controls	EMP Detection Method	Biomarkers for Detection
Chen et al. (2021) [134]	Active	~2–3	15 (42%)	Disease onset (before IVIg)	CD31+, CD54+: Significantly higher compared to healthy control. CD31+, CD105+: Significantly lower when compared to sub-acute timepoint. Quantitative values not reported. (EMPs were normalised to 10,000 events)	2 weeks from disease onset	CD31+, CD54+: Significantly higher compared to healthy control. CD31+, CD105+: Significantly higher when compared to acute timepoint. Quantitative values not reported. (EMPs were normalised to 10,000 events.)	-	-	-	n = 18 CD31+,CD105+: Significantly higher in sub-acute group compared to healthy controls. CD31+, CD54+: Higher in acute and sub-acute phase compared to healthy controls.	Flow cytometry	CD31+, CD54+ and CD31+, CD105+
Nakaoka et al. (2018) [129]	Active	0.3–14	20 (40%)	Time of diagnosis	1.31% (Normalised to total number of particles)	-	-	2–4 weeks after onset of disease.	Below acute levels	-	Healthy: 25 EMP: 0.08% Febrile: 25 EMP:0.09%	Flow cytometry	CD144+/CD42b-
Tian et al. (2016) [118]	In vitro	-	-	-	-	-	-	-	-	-	-	ELISA	CD31, CD62
Shah et al. (2015) [105]	Survivor	Age at study: 11.9 (4.3–32.2) Age at diagnosis: 4.9 (0.18–11.3)	45 (49%)	-	-	-	-	-	-	In KD survivors, AnnexinV: 970 × 10^3^/mL of plasma CD105: 1.60 × 10^3^/mL of plasma (*p* = 0.04) CD62E: 2.87 × 10^3^/mL of plasma CD54: 0.87 × 10^3^/mL of plasma CD106: 0/mL of plasma CD144: 0.32 × 10^3^/mL of plasma CD31: 14.18 × 10^3^/mL of plasma CD42a: 14.04 × 10^3^/mL of plasma	n = 51 AnnexinV: 990 × 10^3^/mL of plasma CD105: 0/mL of plasma CD62E: 3.92 × 10^3^/mL of plasma CD54: 0.97 × 10^3^/mL of plasma CD106: 0/mL of plasma CD144: 0.2 × 10^3^/mL of plasma CD31: 20.59 × 10^3^/mL of plasma CD42a: 24.93 × 10^3^/mL of plasma	Flow cytometry	Annexin V+ and CD105+/CD62E+/CD54+/CD106+/CD144+/CD31+/CD42a-
Ding et al. (2014) [131]	Active	1.9 (0.3–7.5)	12 (42.9%)	unspecified	Absolute values are not reported. All 3 EMPs are significantly elevated in the acute phase when compared to healthy controls but not with febrile control.	unspecified	Absolute values are not reported. All 3 EMPs are significantly elevated in the sub-acute phase when compared to healthy controls but not with febrile control.	unspecified	Absolute values are not reported. All 3 EMPs are significantly elevated at convalescent phase when compared to healthy controls but not with febrile control.	-	Healthy: 28 Febrile: 28	Flow cytometry	CD144+/CD42b−, CD62E+ and CD105+
Tan et al. (2013) [133]	Active	<3 years	Not reported	Within 10 days	n = 20 28.07% (Normalised to 10,000 particles.)	-	-	-	-	-	Healthy: 18 EMP: 11.7% Disease: 18 EMP: 17.2%	Flow cytometry	CD31, CD146
Dou et al. (2013) [130]	KD rabbit model	-	-	-	-	-	-	-	-	-	-	Scanning electron microscope	-
Guiducci et al. (2011) [128]	Active	1.4 (median age)	11 (37%)	Before IVIg	76 × 10^5^/mL plasma.	-	-	1-month follow-up	9 × 10^5^/mL plasma	-	n = 20 45 × 10^5^/mL plasma	Flow cytometry	CD144
Brogan et al. (2004) [135]	In vitro	-	-	-	-	-	-	-	-	-	-	Flow cytometry	CD54, CD106, CD62E, CD62P

## Abbreviations

AECA	anti-endothelial autoantibodies
BLK	B-cell lymphoid kinase
CASP3	caspase-3
CAL	coronary artery lesions
CEC	circulating endothelial cells
cfDNA	cell-free nuclear DNA
CA	coronary artery
CAD	coronary artery disease
DAMP	damage-associated molecular pattern
EC	endothelial cells
EndoMT	endothelial-mesenchymal transition
EMP	endothelial microparticle
EC-cfDNA	endothelial cell-free DNA
ELISA	enzyme-linked immunosorbent assay
FCGR2A	Fc fragment of IgG receptor IIa
GWAS	genome-wide association studies
IVIg	intravenous immunoglobulin
IL	interleukin
iNOS	inducible NOS
KD	Kawasaki disease
MIS-C	multisystem inflammatory syndrome in children
MRP	myeloid-related protein
NLRP3	nucleotide-binding domain and leucine-rich repeat pyrin domain containing 3
NO	nitric oxide
NOS	nitric oxide synthase
NFATs	nuclear factor of activated T-cells
ROS	reactive oxygen species
RAGE	receptor for advanced glycation end-products
SNP	single-nucleotide polymorphisms
TGF-β	transforming growth factor β
VE-Cadherin	vascular endothelial (VE)-cadherin
VEGF	vascular endothelial growth factor
